# The (cost-)effectiveness of a patient-tailored intervention programme to enhance adherence to antihypertensive medication in community pharmacies: study protocol of a randomised controlled trial

**DOI:** 10.1186/s13063-016-1696-3

**Published:** 2017-01-19

**Authors:** Danielle M. van der Laan, Petra J. M. Elders, Christel C. L. M. Boons, Judith E. Bosmans, Giel Nijpels, Jacqueline G. Hugtenburg

**Affiliations:** 1 Department of Clinical Pharmacology and Pharmacy and the Amsterdam Public Health Research Institute, VU University Medical Center, Amsterdam, The Netherlands; 2 Department of General Practice and Elderly Care Medicine and the Amsterdam Public Health Research Institute, VU University Medical Center, Amsterdam, The Netherlands; 3 Department of Clinical Pharmacology and Pharmacy, VU University Medical Center, Amsterdam, The Netherlands; 4 Department of Health Sciences and the Amsterdam Public Health Research Institute, Faculty of Earth and Life Sciences, VU University, Amsterdam, The Netherlands

**Keywords:** Medication non-adherence, Antihypertensive medication, Patient-tailored intervention, Randomised controlled trial

## Abstract

**Background:**

Medication non-adherence is a complex health care problem. Due to non-adherence, substantial numbers of cardiovascular patients benefit from their medication to only a limited extent. In order to improve adherence, a variety of pharmacist-led interventions have been developed. However, even the most effective interventions achieved only a modest positive effect. To be effective, interventions should be targeted at underlying barriers to adherence, developed in a systematic manner and tailored to specific features of a target group and setting. The current paper describes the design of the Cardiovascular medication non-Adherence Tailored Intervention (CATI) study aimed to evaluate the (cost-)effectiveness of a patient-tailored intervention programme in patients using antihypertensive medication.

**Methods:**

The CATI study is a randomised controlled trial that will be performed in 13 community pharmacies. Patients aged 45–75 years using antihypertensive medication and considered non-adherent according to pharmacy dispensing data, as well according to a self-report questionnaire, are eligible to participate. Patients in the intervention condition will receive a patient-tailored, pharmacist-led intervention programme. This programme consists of a structured interview at the pharmacy to identify patients’ barriers to adherence and to counsel patients in order to overcome these barriers. The primary outcome is self-reported medication adherence measured with the MARS-5 questionnaire. Secondary outcome measures are blood pressure, illness perceptions, quality of life and societal costs. A cost-effectiveness analysis and process evaluation will also be performed.

**Discussion:**

This study will provide insight into the (cost-)effectiveness of a patient-tailored, pharmacist-led intervention programme in non-adherent patients using antihypertensive medication. This intervention programme allows community pharmacists to support their patients in overcoming barriers to adherence and improving medication adherence in a structured and patient-tailored manner. An effective intervention will not only enhance medication adherence, but may also improve health outcomes and decrease health care utilisation and costs.

**Trial registration:**

Netherlands Trial Register (identifier: NTR5017), registered on 2 February 2015.

**Electronic supplementary material:**

The online version of this article (doi:10.1186/s13063-016-1696-3) contains supplementary material, which is available to authorized users.

## Background

The World Health Organisation (WHO) provides evidence-based guidelines for the treatment of a variety of disorders, as specified in both pharmacological and non-pharmacological treatment strategies [1]. These strategies as implemented in numerous national guidelines aim to reduce risks, (co)morbidity and mortality [1, 2]. Due to inadequately following pharmacological treatment plans, i.e. medication non-adherence, a subgroup of patients benefits from their medication to only a limited extent. Medication non-adherence is a complex health care problem and defined as the process by which patients take their medication as agreed upon with their prescriber [3]. Causes of non-adherence are patient-, social/economic-, condition-, treatment- or health care system-related [4, 5].

Pharmacological treatment of hypertension can result in a reduced risk of cardiovascular events such as stroke and myocardial infarction [5, 6]. Unfortunately, adherence to antihypertensive medication is often suboptimal and is associated with negative health outcomes, such as cardiovascular events [7–9], higher risk of hospitalisation [8–10] and increased health care costs [8]. A meta-analysis of data of 376,162 patients from 20 studies assessing adherence by using prescription refill data of seven cardiovascular drug classes revealed an estimated non-adherence rate of 43% [11]. Adherence varies depending on drug class with non-adherence rates ranging from 35% for angiotensin II-receptor blockers to 72% for beta-blockers [12].

In order to improve patients’ adherence to medication, a variety of mostly pharmacist-led interventions has been developed. However, reviews summarising the results of a number of studies on the effectiveness of these interventions revealed that in only half of the studies adherence was significantly improved as compared to usual care and that in only a few studies better treatment outcomes were achieved [13–18]. One likely explanation is that most studies did not use a theoretical framework, crucial for understanding the complexities of adherence behaviour. On top of that, most described interventions did not made an effort to apply a patient-tailored approach for identifying the specific causes or barriers for individual patients [5, 19]. Finally, most studies were targeted at the general population rather than at patients non-adherent with their medication.

For this study, the Self-regulation Theory has been chosen as a foundation of the intervention programme. According to this theory patients seek to understand their illness by developing a representation of the illness, its cause, its effects, how long it will last and whether it can be cured or controlled [20, 21]. These illness and treatment representations guide their health behaviour. For instance, if a patient regards his or her illness or risk factor for an illness as a problem, the patient will perform health-related behaviour aimed to solve the problem, e.g. taking medication [20, 22–25]. However, multiple factors influence adherence behaviour [5] and it should be recognised that in this theory these influencing factors can be mediated by patients’ illness and treatment representations.

The Cardiovascular medication non-Adherence Tailored Intervention (CATI) study aims to evaluate the (cost-)effectiveness of a patient-tailored, pharmacist-led intervention (CATI intervention programme) aiming to overcome barriers and improve antihypertensive medication adherence in comparison to usual care. The intervention programme includes identifying factors that influence patients’ adherence behaviour, discussing patient’s illness and treatment representations and suggesting a plan to overcome barriers and improve medication adherence.

## Methods

The CATI study protocol was approved by the Medical Ethics Committee of the VU University Medical Center, Amsterdam (reference 2015/219). Written informed consent will be obtained from all study participants.

### Study design

**Fig. 1:**
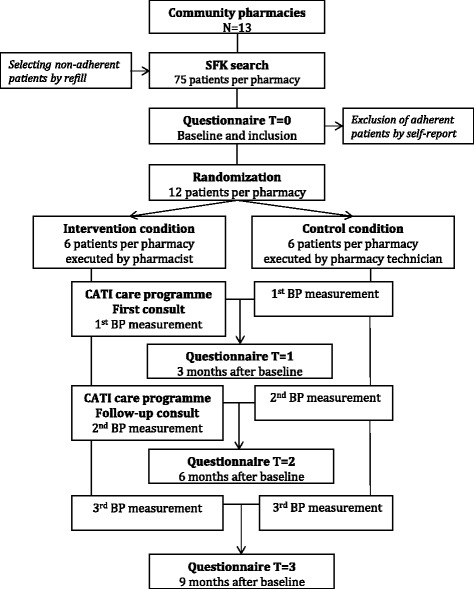
Design of the Cardiovascular medication non-Adherence Tailored Intervention (CATI) study

A parallel-group randomised controlled trial will be performed in 13 community pharmacies including 156 patients (Fig. 1). Patients will be randomly assigned to the intervention or the control condition. Patients in the intervention condition will receive the CATI intervention programme performed by the pharmacist, in addition to usual care (further described below in 'Control condition'). The effectiveness of the CATI intervention programme will be measured during a 9-month follow-up. See Additional file 1 for an overview of the Standard Protocol Items: Recommendations for Interventional Trials (SPIRIT) 2013 checklist items [26]. See Additional file 2 for the SPIRIT diagram of the trial procedure.

### Study population and setting

Patients will be recruited from community pharmacies located in urban and rural areas in The Netherlands. Participating pharmacies must have a subscription to the Foundation for Pharmaceutical Statistics (SFK), which is developed by the Royal Dutch Pharmacists Association. SFK registers information on dispensing drugs and will be used for the selection of eligible patients. Patients aged 45–75 years using antihypertensive medication and indicating to have hypertension by self-report are eligible to participate. Antihypertensive medication includes beta-blockers, calcium antagonists, diuretics, angiotensin converting enzyme (ACE) inhibitors and angiotensin II-receptor antagonists. Only patients non-adherent with their antihypertensive medication according to pharmacy dispensing data, as well according to a self-report questionnaire, are eligible. Exclusion criteria are the inability to speak, read and write Dutch, and the use of special medication-intake supporting services provided by the pharmacy, i.e. repeat dispensing and pill packaging.

### Inclusion procedure

To be included in the intervention or the control condition a two-step approach will be applied.

#### Step 1

Eligible patients will be identified by a SFK database search which provides information on dispensed medication. For each class of antihypertensive medication SFK calculates a score by dividing the number of covered days by the length of the therapy window. The number of covered days is calculated by summating the days in the therapy window covered by usage periods which are corrected for overlapping supplies. A score lower than 80% is considered non-adherent. SFK provides a list of patients non-adherent in refilling at least one of the classes of antihypertensive medication during the last 6 months. The researcher will check this list in order to prevent selecting patients who are most likely falsely identified as non-adherent due to switching drugs or hospital stay.

#### Step 2

Patients identified as non-adherent in refilling antihypertensive medication will be invited to fill out a questionnaire. This baseline questionnaire includes an Informed Consent Form and the five-item Medication Adherence Report Scale (MARS-5) questionnaire [27]. The MARS-5 questionnaire assesses self-report medication intake behaviour by five questions rated on a five-point scale and ranges from 5 to 25 points. Patients with a score of below 25 points are considered non-adherent.

Patients who are willing to participate and identified as non-adherent with their antihypertensive medication according to pharmacy dispensing data, as well according to the self-report questionnaire, will be included in the study. Patients identified as non-adherent with only one non-adherence measure will not be included. Patients not willing to participate will be asked to fill out a short questionnaire assessing demographic variables and reasons for their nonparticipation.

### Randomisation

To each eligible patient an unique serial number will be assigned. Subsequently, a member of the research team will perform the randomisation process using blocking [28] with a block size of 4, a random cell selection a 1:1 allocation ratio. A list of patients randomised to the intervention or the control condition will be handed to the pharmacist and pharmacy technician, respectively. Blinding of the participants, pharmacists and pharmacy technicians to treatment allocation is not possible due to the nature of the intervention.

### Intervention condition

Patients in the intervention condition will receive the CATI intervention programme in addition to usual care. The programme is a patient-tailored, pharmacist-led, theory-based intervention programme to enhance adherence with the use of antihypertensive medication. It consists of two steps. First, the patients’ barriers to adherence will be identified, and second, tailored information and advice on the identified barriers and specific needs of the patient will be provided. The Self-regulation Theory suggests that patients make an illness and treatment representation which guides their health behaviour. By providing tailored information to the patient regarding the potential risks of high blood pressure and the need for treatment, it is expected to increase patients’ understanding and perceived need to be adherent with their antihypertensive medication. The programme will be executed by the pharmacist and includes two visits to the pharmacy.

#### First consult

The pharmacist invites the patient for a consult in the pharmacy. The consult starts by interviewing the patient using a semistructured interview guide, called the Quick Barrier Scan (QBS). The aim of the QBS is to explore patients’ barriers to adherence to medication assessed by means of 12 questions, for instance concerning knowledge, side effects, forgetfulness, difficulties with medication intake and lack of motivation. Patients will also be asked about their own ideas of intake barriers. Based upon the identified barrier(s) an intervention module will be selected according to the Tailored Intervention Guide (TIG). The TIG provides an overview of five intervention modules aiming to overcome different barriers to adherence. Each assessed barrier in the QBS conforms to an intervention module in the TIG. If no clear barrier can be identified with the QBS, the first intervention module will be conducted. The five intervention modules include: (1) Providing Information, (2) Providing Tools, (3) Dealing with Side Effects, (4) Overcoming Practical Problems and (5) Diminishing Negative Beliefs. Each intervention module consists of three components. During the first component, the patient will be provided with information regarding the representation and potential risks of high blood pressure, the use of antihypertensive medication and living a healthy lifestyle. During the second component, the pharmacist will give the patient tailored advice on overcoming the identified barriers. The content of the second component is different for each intervention module. For instance, possible recommendations of the second intervention module (Providing Tools) are connecting intake with daily recurring activities or the use of a pill box or medication reminder alarm. Recommendations of the third intervention module (Dealing with Side Effects) are to balance patients’ advantages and disadvantages of medication use; whereas examples of recommendations of the fourth intervention module (Practical Problems) are to register a patient for an intake-supporting service, i.e. pill packaging. During the third component, a summary is made from the information and advice discussed by the pharmacist in order to provide the patient with a short overview of the first consult. This overview can be used as an action plan. The intervention programme and materials are partly based upon the promising results of a previous study in which the feasibility of a modular intervention programme in patients with diabetes was evaluated [29]. On top of that, the intervention materials (the QBS and the TIG) were further developed based upon our own research including a systematic literature review and a cross-sectional study (manuscripts in preparation). The systematic review aimed to provide an overview of the factors influencing antihypertensive medication adherence. The cross-sectional study used a questionnaire to assess barriers to adherence in a Dutch population using cardiovascular medication. These results provided a complete overview of potential barriers to medication adherence which should be incorporated in the intervention materials. Lastly, the intervention materials were approved by an expert panel including a pharmacist, a general practitioner and a sociologist.

#### Follow-up consult

Approximately 3 months after the first consult a follow-up consult will be planned with the patient. The purpose of this follow-up consult is to discuss patients’ implementation of and patients’ experiences with the action plan. If needed, the developed action plan of the first consult will be adjusted based upon patients’ experiences.

During the first consult, the follow-up consult and during an additionally planned visit to the pharmacy, patients’ systolic and diastolic blood pressure will be measured with an automatic sphygmomanometer according to a protocol.

### Training

Participating pharmacists will follow a 1-day training session. The training session comprises a theoretical and a practical part. During the theoretical part, background information on medication non-adherence will be discussed and instructions will be given on study design, protocol and intervention materials. An experienced communication lecturer will provide information and instructions on how to communicate with patients. During the practical part, the pharmacists will practise with communication skills and intervention materials in a patient-pharmacist role-playing setting. Before and after the training session the pharmacists will fill out a form to assess the potential improvement of their knowledge and competencies.

### Control condition

Patients in the control condition will receive usual care according to the Dutch guidelines of the Royal Dutch Pharmacists Association [30]. In summary, the care according to these guidelines consist of checking and dispensing of prescribed drugs, providing instructions on medication use, and providing information about intended effects and possible side effects, during first and second dispensing.

### Primary outcome measure

Self-reported medication adherence will be assessed with the MARS-5 questionnaire at baseline and after 3, 6 and 9 months [27]. The MARS-5 questionnaire comprises five statements of adherence-related behaviour rated on a five-point scale, where 1 = ‘always’, 2 = ‘often’, 3 = ‘sometimes’, 4 = ‘rarely’ and 5 = ‘never’. Scores for each item will be summated to give a total score, ranging from 5 to 25 points. A score below 25 points is considered non-adherent. The questionnaire distinguishes intentional non-adherence and unintentional non-adherence. The statements assessing intentional non-adherence are ‘I alter the dose of my medicines’, ‘I stop taking my medicines for a while’, I decide to miss out on a dose of my medicines’ and ‘I take less of my medicines than instructed’. The statement assessing unintentional non-adherence is ‘I forget to take my medicines’. The MARS-5 questionnaire is easy to use and shows to be a valid and reliable tool for measuring patients’ non-adherence to medication across different illnesses including asthma, diabetes and hypertension [27, 31–33]. The internal reliability (Cronbach’s *α*) ranged from 0.67 to 0.90; test-retest reliability (Pearson’s *r*) was 0.97; and concurrent validity (Pearson’s *r*) with the Morisky Medication Adherence Scale [34] was 0.62. The MARS-5 questionnaire was selected because it is a sufficiently-validated and easy-to-use tool. Furthermore, in addition to measuring the extent of non-adherence, it can distinguish between patients with intentional and unintentional medication non-adherence.

### Secondary outcome measures

Secondary outcomes include patients’ attitudes and beliefs towards medication, quality of life (QoL), illness perceptions, costs and systolic and diastolic blood pressure.

#### Attitudes and beliefs towards medication

Patients’ attitudes and beliefs towards medication will be assessed with the specific Beliefs about Medicines Questionnaire (BMQ) [35, 36] at baseline and after 3, 6 and 9 months. The BMQ Specific can be subdivided into the BMQ Specific Necessity and the BMQ Specific Concern. The BMQ Specific Necessity measures the patients’ beliefs of the necessity of taking the medication and the BMQ Specific Concern measures the patients’ concerns about taking medication. Both subscales range from 5 to 25. The BMQ Necessity-Concern differential can be calculated as the difference between the Concern and Necessity subscales. The BMQ specific subscales can be used to categorise patients into four attitudinal groups: accepting (high necessity, low concerns), ambivalent (high necessity, high concerns), indifferent (low necessity, low concerns) and sceptical (low necessity, high concerns). Research shows evidence on good reliability and acceptable validity of the BMQ scales and supports its use as a research tool when investigating patients’ beliefs about medication [35–37]. The internal reliability (Cronbach’s *α*) for both the Concern and Necessity subscales was 0.83. When assessing the validity of the BMQ with the MARS-5, significant predictive correlations were found for both the Concern and Necessity subscales [37]. The measurement of patient’s beliefs about medication is important because it provides insight into the mechanism by which medication beliefs might influence medication adherence.

#### Quality of life

Patients’ QoL will be assessed using both the valid and reliable 12-Item Short Form (SF-12) questionnaire [38] and the valid and reliable EuroQol (EQ-5D-5L) questionnaire [39, 40] at baseline and after 3, 6 and 9 months. The SF-12 questionnaire consists of 12 questions covering eight dimensions of health, i.e. general health perception, physical functioning, limitations due to physical health problems, bodily pain, vitality, social functioning, emotional functioning and general mental health. The eight domains can produce two summary scores for physical health and mental health. The EQ-5D-5L questionnaire is a standardised instrument measuring five health dimensions (mobility, self-care, usual activities, pain/discomfort and anxiety/depression) with five severity levels each [39]. The EQ-5D-5L questionnaire health states will be converted into utility scores using the Dutch tariff [41]. Utility scores reflect the desirability of a health state and are anchored at 0 (‘death’) and 1 (‘perfect health’). Quality-adjusted life years will be calculated by multiplying the utility of a particular health state by the time spent in that health state using the area-under-the-curve method. Transitions between health states are considered linear.

#### Illness perceptions

The Brief Illness Perceptions Questionnaire (Brief IPQ) is a nine-item scale designed to assess patients’ cognitive and emotional representations of illness [42]. All items except the causal question are rated on 0 to 10 scale. Five items assess cognitive illness representations: consequences, timeline, personal control, treatment control and identity. Two items assess emotional representations: concerns and emotions. One item assesses illness comprehensibility. The causal representation question asks listing the three most important causal factors of illness. The reliable and valid Brief IPQ is measured at baseline and after 3, 6 and 9 months.

#### Costs

Costs will be measured from a societal perspective using adapted versions of the iMTA Medical Cost Questionnaire (iMCQ) [43] and the iMTA Productivity Cost Questionnaire (iPCQ) [44] including costs of health care utilisation, informal care, and work absenteeism and presenteeism. Medication use will be retrieved from the patient’s pharmacy. If available, Dutch guideline prices will be used to value resource use. Medication use will be valued using prices set by the Royal Dutch Pharmacists Association. Lost productivity costs will be calculated according to the friction cost approach using Dutch mean incomes [45]. According to the friction cost approach a sick employee is replaced after a certain amount of time (the friction period) after which there are no further lost productivity costs [46]. All costs will be adjusted to the year in which most data is collected using consumer price indices.

#### Blood pressure

Systolic and diastolic blood pressure is a clinical secondary outcome and will be measured with an automatic sphygmomanometer in both the intervention and the control conditions according to a standardised protocol. In the intervention condition blood pressure will be measured by the pharmacists at the start of the first consult, at the start of the follow-up consult and at a final pharmacy visit. In the control condition blood pressure will be measured by the pharmacy technician during three pharmacy visits at baseline and after 3 and 6 months. At each visit blood pressure will be measured three times, each 2 min apart, in a seated position.

### Pilot study

Based on the results of a pilot study the CATI intervention programme was improved and finalised. Ten patients from three community pharmacies participated in the pilot study. These participants filled out the baseline questionnaire and visited the pharmacy for the first consult. Logistics, questionnaires, use of study protocol and intervention materials, and the feasibility of the intervention programme were evaluated. After the pilot study some aspects of the intervention programme were changed, for instance putting more emphasis on the follow-up consult and development of an action plan; simplifying the structure and use of the intervention materials; and adapting logistic aspects to increase feasibility.

### Statistical analyses

Descriptive statistics will be used to characterise the study population. Dropout and loss to follow-up will also be described. The effect analyses will be performed according to the ‘intention-to-treat’ and ‘per-protocol’ principles. Linear and logistic mixed-model analyses will be used to assess the effect of the intervention programme by comparing the differences between the intervention and the control conditions. Mixed-model analysis is needed in order to take into account clustering on pharmacy level and repeated measurements in one patient. Adjustments will be made for possible confounders such as gender, age and education level. The researcher will be blinded during data analyses.

### Sample size

A sample size calculation for proportions has been performed. The sample size is based upon the difference in percentage of adherent participants over 9 months of follow-up between the intervention and the control conditions. A difference of 20% is considered relevant. At baseline, all participants are non-adherent as measured with the self-report adherence questionnaire. The expected percentage of adherent participants (MARS-5 = 25) at the end of the study is 30% for the intervention condition and 10% for the control condition. When using an alpha of 0.05 and a beta of 0.20, a group size of 60 is sufficient, taking the clustered and longitudinal design into account. To adjust for a loss to follow-up of approximately 30%, we will include 156 patients.

### Process evaluation

The process evaluation assesses the extent to which the CATI intervention programme is performed according to study protocol and gives insight into barriers and facilitators in executing the different components of the intervention programme. Data will be collected on time that is spent by the pharmacists in executing the intervention programme and the intervention modules that are selected and performed. Data on these topics will be collected using an administration form. In addition, semistructured interviews will be held with the participating pharmacists at the end of the study in order to record experiences and opinions on the intervention programme. The presence of possible contamination will also be assessed by interviewing the pharmacy technicians. Furthermore, after the follow-up consult participants will fill out a questionnaire to evaluate the different components of the intervention programme.

### Economic evaluation

Both a cost-effectiveness analysis and a cost-utility analysis will be performed according to the intention-to-treat principle. Missing cost and effect data will be imputed using Multiple Imputation by Chained Equations (MICE) [47]. To account for the skewed distribution of costs Predictive Mean Matching will be used in the MICE procedure. The number of imputed datasets will be increased until the loss of efficiency is less than 5% [48]. The results of the imputed datasets will be pooled according to Rubin’s rules. Bivariate regression analyses will be used to estimate cost and effect differences while adjusting for potential confounders and maintaining the correlation between costs and effects. Incremental Cost-Effectiveness Ratios (ICERs) will be calculated by dividing the mean difference in total societal costs by the mean difference in effects. Bias-corrected and accelerated bootstrapping with 5000 replications will be used to estimate 95% confidence intervals around cost and effect differences, and to estimate the uncertainty surrounding the ICERs which will be graphically presented on a cost-effectiveness plane [49]. Cost-effectiveness acceptability curves will be estimated in which the probability that the intervention programme is cost-effective in comparison to usual care is plotted on the y-axis, while the willingness-to-pay per incremental unit of effect is plotted on the x-axis [50].

## Discussion

This study is expected to add evidence to the (cost-)effectiveness of a patient-tailored, theory-driven, pharmacist-led intervention programme as compared to usual care in patients using antihypertensive medication. The intervention programme includes a variety of tools to support patients to overcome barriers to adherence, thereby improving medication adherence. It is expected that patients will be encouraged to adjust their adherence behaviour. This will be achieved by learning from the provided information, following the pharmacists’ advice and by using supporting aids. In addition, performing an action plan and discussing the use and results of the plan at a follow-up visit may increase these achievements. Moreover, the personal attention of the pharmacist towards the patient is expected to be a stimulating factor for behavioral change.

A strength of this study is the theory-driven and patient-tailored approach of the intervention programme. All patients have their own medication intake behaviour, experience their own problems and barriers to adherence and have their own needs. By focussing on identifying patients’ barriers in the initial phase and subsequently addressing these barriers by providing specific information and advice to patients, the patient-tailored approach is applied. Moreover, encouraging patients to self-regulate their medication intake behaviour by overcoming specific barriers will lead to the improvement of medication adherence. In addition, this intervention programme has been developed for patients non-adherent with their antihypertensive medication, and will not unnecessarily be addressed to the general population of antihypertensive medication users. Another strength of the study is the execution of a pilot study which resulted in improving and finalising the intervention programme. The study will be conducted in daily clinical practice. This increases the feasibility of implementing the CATI intervention programme in a community pharmacy setting on a large scale. Furthermore, alongside the study a process evaluation will be performed. This will enable us to assess the implementation fidelity and add to a careful interpretation of the results of the study.

The MARS-5 questionnaire has been selected as the primary outcome for this study. Two studies showed poor sensitivity results of the MARS-5 questionnaire [51, 52]. This could be a limitation of this study. An explanation for these results could be the use of the Medication Possession Ratio as a reference standard. This method calculates the extent to which patients refill their medication, rather than calculating the extent to which patients take their medication. Despite these results, the MARS-5 questionnaire has several advantages. First, the questionnaire performs well on other psychometric properties. Second, the response scale allows patients to be graded in terms of the frequency with which they engage in non-adherent behaviours. Third, the questionnaire can distinguish between patients with intentional and unintentional medication non-adherence. Lastly, this questionnaire is relatively short and easy to use. Another limitation of this study is that, due to the nature of the intervention, it is not possible to blind the researcher, patients, pharmacists or pharmacy technicians to the group allocation of participants. To minimise possible ascertainment bias before data collection participants were informed about being randomised into one out of two programmes, with either three blood pressure measurements or two consults and three blood pressure measurements. Moreover, to minimise possible ascertainment bias after data collection, the researcher will be blinded during data analyses. A final limitation of this study is that due to performing the randomisation at the individual level both the intervention and the control conditions will be conducted in one pharmacy which can lead to contamination. To minimise contamination within one pharmacy, the intervention programme and blood pressure measurements will be performed by a pharmacist, whereas the blood pressure measurements in the control condition will be performed by a pharmacy technician; previously, pharmacy technicians had been instructed to perform no activities other than usual care during the three blood pressure measurement visits.

If the CATI intervention programme is feasible and effective, it could potentially be extrapolated to other groups of patients in whom medication non-adherence is also a problem. The information gained from this study may prove useful for policy-makers, health care providers, and researchers who are in the process of improving adherence with the use of (cardiovascular) medication.

### Trial status

The inclusion of patients and the collection of data started in March 2016. The inclusion of patients ended in the third quarter of 2016. Results will be expected in 2017.

## Additional files

Additional file 1: Standard Protocol Items: Recommendations for Interventional Trials (SPIRIT) 2013 Checklist. (DOC 100 kb)
Additional file 2: SPIRIT diagram. (DOC 49 kb)

## Abbreviations

BMQ: Beliefs about Medicines Questionnaire
BP: Blood pressure
Brief IPQ: Brief Illness Perceptions Questionnaire
CATI: Cardiovascular medication non-Adherence Tailored Intervention
MARS-5: Medication Adherence Report Scale
QBS: Quick Barrier Scan
QoL: Quality of Life
SFK: Foundation for Pharmaceutical Statistics
SPIRIT: Standard Protocol Items: Recommendations for Interventional Trials
TIG: Tailored Intervention Guide
