# Supplementary material for: The (cost-)effectiveness of a patient-tailored intervention programme to enhance adherence to antihypertensive medication in community pharmacies: study protocol of a randomised controlled trial
Source: Trials. 2017 Jan 19;18:29. doi: 10.1186/s13063-016-1696-3 (PMC5244518; doi:10.1186/s13063-016-1696-3)
Supplement: Additional file 2: — SPIRIT diagram. (DOC 49 kb) [file 13063_2016_1696_MOESM2_ESM.doc]

Figure 2. SPIRIT diagram.

|  | Study period | | | | | |
| --- | --- | --- | --- | --- | --- | --- |
|  | Enrolment | Allocation | Post-allocation | | | |
| TIMEPOINT | *Pre-intervention* | *Pre-intervention* | *T0* | *T1* | *T2* | *T3* |
| ENROLMENT: |  |  |  |  |  |  |
| *Eligibility screen 1* | X |  |  |  |  |  |
| *Eligibility screen 2* | X |  |  |  |  |  |
| *Informed consent* | X |  |  |  |  |  |
| *Baseline questionnaire* | X |  |  |  |  |  |
| *Allocation* |  | X |  |  |  |  |
| INTERVENTIONS: |  |  |  |  |  |  |
| *Intervention condition* |  |  |  |  |  |  |
| ASSESSMENTS: |  |  |  |  |  |  |
| *Demographic variables* |  |  | X |  |  |  |
| *MARS-5* |  |  | X | X | X | X |
| *BMQ* |  |  | X | X | X | X |
| *SF-12, EQ-5D-5L* |  |  | X | X | X | X |
| *Brief IPQ* |  |  | X | X | X | X |
| *iMCQ, iPCQ* |  |  |  | X | X | X |
| *Blood pressure* |  |  | X | X | X |  |
